# Mechanical and Surface Behavior of Contemporary Restorative Materials Exposed to Topical Hemostatic Agents After Artificial Aging: An In Vitro Study

**DOI:** 10.3390/polym18172111

**Published:** 2026-08-31

**Authors:** İlhan Kaya, Sena Nur Yıldırım, Akif Demirel

**Affiliations:** 1 Department of Oral and Maxillofacial Surgery, Faculty of Dentistry, Bursa Uludağ University, 16120 Bursa, Turkey; 2 Department of Pediatric Dentistry, Faculty of Dentistry, Ankara University, 06560 Ankara, Turkey; snuryildirim@ankara.edu.tr (S.N.Y.); akifdemirel@ankara.edu.tr (A.D.)

**Keywords:** pediatric dentistry, hemostatic agents, restorative dental materials, fracture-load, surface roughness, thermocycling

## Abstract

Hemostatic agents are frequently used during pediatric dental procedures and may inadvertently contact restorative materials. This study evaluated the fracture-load and surface roughness of contemporary pediatric restorative materials following exposure to different surgical hemostatic agents after artificial aging. A total of 360 disc-shaped specimens were prepared from three pediatric restorative materials (composite, compomer, and glass hybrid), with separate sets of 180 specimens allocated to fracture-load and surface roughness assessment. For each outcome, specimens were exposed to ferric sulfate, tranexamic acid, or Ankaferd Blood Stopper for 20 s and subjected to either 5000 or 10,000 thermocycles. Data were analyzed using three-way factorial general linear models (α = 0.05). Composite exhibited higher fracture-load values than compomer and glass hybrid (both adjusted *p* < 0.001) and lower surface roughness than glass hybrid (adjusted *p* = 0.004). Transamine and Ankaferd showed higher fracture-load values than ferric sulfate (adjusted *p* = 0.003 and *p* = 0.020, respectively). All hemostatic-agent comparisons for surface roughness were significant (all adjusted *p* < 0.001), with Transamine showing the lowest and ferric sulfate the highest values. Specimens subjected to 10,000 thermocycles exhibited lower fracture-load and higher surface roughness than those subjected to 5000 thermocycles (both *p* < 0.001). A significant hemostatic agent × thermocycling interaction was observed for surface roughness. Fracture-load and surface roughness differed according to restorative material, surgical hemostatic agent, and thermocycling protocol. Selecting an appropriate surgical hemostatic agent may help preserve the mechanical and surface characteristics of pediatric restorative materials following artificial aging.

## 1. Introduction

Direct restorative dental materials play a fundamental role in contemporary pediatric dentistry by restoring function, esthetics and structural integrity in teeth affected by dental caries or trauma [1,2,3]. Advances in restorative material technology have expanded the range of available materials, including resin-based composites, compomers and glass hybrid restorative systems, each possessing distinct mechanical and surface characteristics. Although these materials have demonstrated satisfactory clinical performance, their long-term success depends not only on their intrinsic properties but also on the clinical conditions encountered during restorative procedures [1,2,3,4,5,6].

Achieving adequate moisture and bleeding control is an essential prerequisite for successful dental and oral surgical procedures. In dental practice, bleeding may occur during a wide range of clinical procedures, including restorative treatments, subgingival cavity management, pulp therapy, periodontal interventions, tooth extractions, and other oral surgical procedures [1,7]. Therefore, local hemostatic agents are frequently used in both restorative and surgical dental procedures to obtain a clean operating field, improve clinical visibility, facilitate treatment, and achieve effective perioperative or postoperative hemostasis when necessary [7,8]. Among the available agents, ferric sulfate has been widely used in dentistry for several decades because of its effective hemostatic action, particularly in restorative dentistry and pediatric pulp therapy [7,8,9]. More recently, Ankaferd Blood Stopper, a plant-derived hemostatic agent, has emerged as an alternative for controlling bleeding during various dental and oral surgical procedures [10,11,12,13]. In addition, tranexamic acid, a synthetic antifibrinolytic agent extensively used in medicine, has recently attracted increasing interest in dentistry as an adjunctive local hemostatic agent for oral surgical procedures, particularly tooth extractions and the management of patients receiving antithrombotic therapy [14,15].

Although these hemostatic agents provide effective bleeding control, their accidental contact with restorative materials during clinical procedures is often unavoidable. Recent evidence indicates that contamination of the operative field with blood or the use of hemostatic agents during adhesive procedures may compromise restorative outcomes, emphasizing the importance of proper contamination control [16,17]. Previous investigations have primarily focused on the influence of hemostatic agents on dentin bonding performance and adhesive strength, particularly for ferric sulfate, demonstrating that residual contamination may adversely affect adhesion if not adequately removed. Experimental and review studies have shown that ferric sulfate contamination may alter dentin surface morphology, interfere with adhesive infiltration, and reduce the bond strength of certain adhesive systems, especially when decontamination is inadequate [7,18,19]. However, considerably less attention has been paid to the direct effects of hemostatic agents on the physical and mechanical properties of restorative materials, such as surface roughness and fracture-load, despite their potential influence on the long-term clinical performance of restorations [17].

Surface roughness and fracture-load are among the most clinically relevant properties affecting the longevity of restorative materials [20,21,22,23,24]. Increased surface roughness promotes plaque accumulation, bacterial adhesion, discoloration, and surface degradation, whereas reduced fracture-load increases the susceptibility of restorations to bulk fracture and clinical failure under occlusal loading [20,25]. Furthermore, restorative materials are continuously exposed to thermal fluctuations in the oral environment, making artificial aging an important component of in vitro investigations to better simulate clinical service conditions [26].

To the best of our knowledge, no previous study has comparatively evaluated the effects of tranexamic acid, Ankaferd Blood Stopper and ferric sulfate on the surface roughness and fracture-load of different pediatric restorative materials following artificial aging with different thermocycling protocols. Therefore, the aim of the present in vitro study was to compare the surface roughness and fracture-load values of a compomer, a microfilled hybrid composite resin, and a glass hybrid restorative material following exposure to three different hemostatic agents and two thermocycling regimens. The null hypotheses were that fracture-load and surface roughness values would not differ according to restorative material, hemostatic agent, or thermocycling protocol, and that no interactions would occur among these factors.

## 2. Materials and Methods

### 2.1. Research Design and Reporting

The present study was designed as an assessor-blinded, in vitro experimental study to evaluate the effects of different hemostatic agents and artificial aging protocols on the surface roughness and fracture-load of commonly used pediatric dental restorative materials. The experimental design consisted of three restorative materials, three hemostatic agents, and two thermocycling protocols, resulting in a 3 × 3 × 2 factorial design. Surface roughness and fracture-load were evaluated using separate sets of specimens; therefore, no specimen was subjected to more than one outcome assessment. The primary objective of this factorial design was to compare the relative mechanical and surface characteristics of restorative materials exposed to three different topical hemostatic agents under two artificial-aging conditions. Because the prespecified research question focused on comparisons among hemostatic-agent-exposed groups rather than on determining the absolute effect of hemostatic-agent exposure versus no exposure, an untreated control group was not incorporated into the original experimental design.

A total of 360 independent disc-shaped specimens were prepared, including 180 specimens for surface roughness analysis and 180 specimens for fracture-load testing. All restorative procedures and hemostatic agent applications were performed by the same investigator (İ.K.) to ensure procedural standardization, whereas the outcome assessments were carried out by blinded examiners (A.D. and S.N.Y.). The study protocol was conducted in accordance with the checklist for reporting in vitro studies (CRIS) guidelines to enhance the methodological quality, transparency, and reproducibility of in vitro dental research [27].

### 2.2. Sample Size Calculation

An a priori power analysis was performed using G*Power software (Version 3.1.9.7; Heinrich Heine University, Düsseldorf, Germany) to calculate the required sample size for each primary outcome measure (surface roughness and fracture-load). The experimental layout followed a 3 × 3 × 2 factorial design, incorporating three restorative materials, three hemostatic agents, and two thermocycling conditions (yielding 18 experimental groups). With an assumed medium effect size (f = 0.25), a significance level (α) of 0.05, a numerator degree of freedom of 2, and a desired statistical power (1-β) of 0.80, the power analysis yielded a total minimum sample size of 158 specimens (actual power = 80.13%). To maintain equal allocation across all 18 experimental groups, this requirement was adjusted to a minimum of 9 specimens per group (*N* = 162). To further enhance statistical power, account for potential procedural variations, and ensure methodological robustness, the sample size was rounded up to 10 specimens per subgroup (*n* = 10). Consequently, 180 independent specimens were allocated to each outcome assessment, resulting in a total of 360 specimens for the overall study. Within each outcome set, specimens were randomly assigned to the hemostatic-agent and thermocycling groups. Random allocation was performed using a computer-generated randomization sequence.

### 2.3. Restorative Dental Materials

Three restorative materials representing different classes of direct pediatric dental restorative materials commonly used in pediatric dentistry were included in the study: a polyacid-modified composite resin (Compomer; Dyract XP, Dentsply DeTrey GmbH, Konstanz, Germany), a microfilled hybrid resin composite (G-ænial Posterior, GC Corporation, Tokyo, Japan), and a glass hybrid restorative system (EQUIA Forte HT, GC Corporation, Tokyo, Japan). These materials were selected because of their widespread clinical use in pediatric restorative dentistry and their distinct compositions and mechanical characteristics. All materials were handled and polymerized strictly according to the manufacturers’ instructions to ensure standardized specimen preparation and minimize technique-related variability. The material classes, manufacturers, and principal chemical components of the restorative materials are summarized in Table 1.

### 2.4. Specimen Preparation

**Figure 1:**
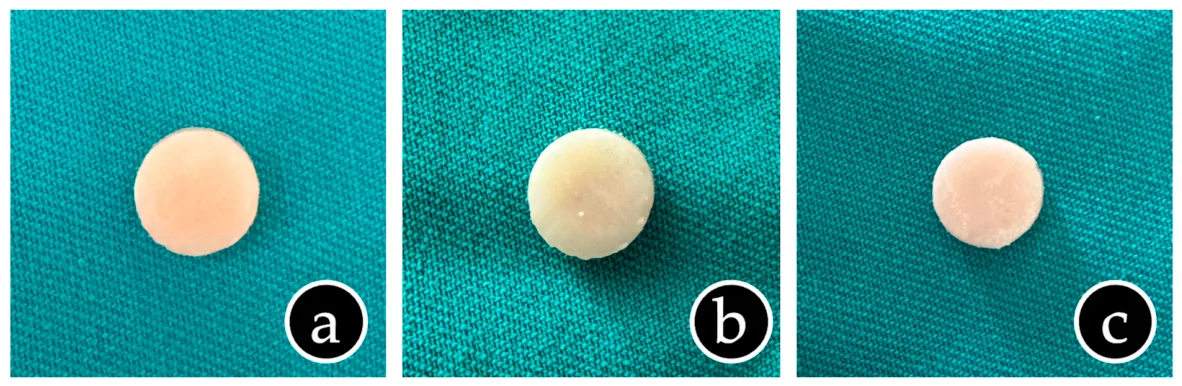
Representative disc-shaped specimens prepared from the three restorative materials evaluated in the study: (**a**) compomer (Dyract XP); (**b**) microfilled hybrid composite resin (G-ænial Posterior); and (**c**) glass hybrid restorative system (EQUIA Forte HT).

Disc-shaped specimens measuring 6 mm in diameter and 2 mm in thickness were fabricated from each restorative material using stainless-steel molds. A total of 360 specimens were prepared, including 120 specimens from each restorative material, with separate specimen sets allocated for surface roughness and fracture-load testing. The compomer (Dyract XP) and microfilled hybrid composite resin (G-ænial Posterior) were placed into the molds in a single increment. A transparent Mylar strip was positioned over the restorative material, followed by a glass microscope slide to obtain a flat and standardized surface while minimizing the oxygen inhibition layer. The specimens were light-cured for 20 s using an LED light-curing unit (Woodpecker i-LED Plus, Guilin Woodpecker Medical Instrument Co., Guilin, China; 1000 mW/cm^2^) according to the manufacturers’ instructions. For the glass hybrid restorative system (EQUIA Forte HT), the encapsulated material was activated and mixed according to the manufacturer’s instructions before placement into the molds. Following the initial setting reaction, the specimen surfaces were finished using Sof-Lex™ coarse polishing discs (3M ESPE, St. Paul, MN, USA). Subsequently, EQUIA Forte Coat (GC Corporation, Tokyo, Japan) was applied to the specimen surface and light-cured for 20 s using the same LED curing unit according to the manufacturer’s instructions (Figure 1). After specimen fabrication, all specimens were carefully removed from the molds and visually inspected for defects such as voids, surface irregularities, or marginal imperfections. Defective specimens were discarded and replaced to maintain the predetermined sample size. All specimens were then stored individually in distilled water at 37 °C for 24 h before allocation to the experimental groups.

### 2.5. Definition of the Study Groups

Separate specimen sets were prepared for the surface roughness and fracture-load analyses. For each outcome, 180 independent specimens were fabricated, comprising 60 specimens from each restorative material. Accordingly, each experimental phase consisted of three restorative materials, three hemostatic agents, and two thermocycling protocols, resulting in a 3 × 3 × 2 factorial design. For each restorative material and outcome, specimens were randomly allocated in equal numbers to six experimental groups (*n* = 10 per group) according to the assigned hemostatic agent and thermocycling protocol.

The experimental groups were defined as follows:

Group 1: Transamine (50 mg/mL) + 5000 thermocycles

Group 2: Transamine (50 mg/mL) + 10,000 thermocycles

Group 3: Ankaferd Blood Stopper Dental Form + 5000 thermocycles

Group 4: Ankaferd Blood Stopper Dental Form + 10,000 thermocycles

Group 5: Astringedent^®^ (15.5% ferric sulfate) + 5000 thermocycles

Group 6: Astringedent^®^ (15.5% ferric sulfate) + 10,000 thermocycles

The same grouping strategy was applied independently to the compomer, microfilled hybrid composite resin, and glass hybrid restorative materials. Consequently, each outcome assessment included 18 experimental subgroups (3 restorative materials × 6 experimental groups), with 10 independent specimens allocated to each subgroup, resulting in a total of 180 specimens per outcome and 360 specimens for the entire study.

### 2.6. Application of Hemostatic Agents

Following specimen preparation and storage in distilled water at 37 °C for 24 h, the specimens were assigned to their respective experimental groups according to the type of hemostatic agent and thermocycling protocol. Three commercially available hemostatic agents were evaluated in this study: Transamine^®^ (tranexamic acid, 50 mg/mL; Teva Pharmaceuticals, Istanbul, Türkiye), Ankaferd Blood Stopper^®^ Dental Form (AND Co., Istanbul, Türkiye), and Astringedent^®^ (15.5% ferric sulfate; Ultradent Products Inc., South Jordan, UT, USA). The assigned hemostatic agent was applied to the entire surface of each specimen using a disposable microbrush and maintained in contact with the restorative material surface for 20 s to simulate accidental clinical contact during restorative procedures. At the end of the application period, each specimen was rinsed with the dental unit air-water spray (tap water) for 5 s to remove any residual hemostatic agent. The specimens were then subjected to the assigned artificial aging protocol. All applications were performed by the same investigator under standardized laboratory conditions.

### 2.7. Artificial Aging and Thermocycling

Following the application of the hemostatic agents, the specimens were subjected to artificial aging according to their assigned experimental groups. Half of the specimens in each hemostatic agent group underwent 5000 thermocycles, while the remaining half underwent 10,000 thermocycles. Thermocycling was performed using a thermocycling device (SD Mechatronik GmbH, Feldkirchen-Westerham, Germany) between water baths maintained at 5 ± 2 °C and 55 ± 2 °C, with a 30 s dwell time in each bath and a 10 s transfer time between baths. Upon completion of the assigned thermocycling protocol, the specimens were immediately subjected to either surface roughness or fracture-load testing according to their predetermined experimental allocation.

### 2.8. Surface Roughness Analysis

Surface roughness analysis was performed using a contact profilometer (Perthometer M2, Mahr GmbH, Göttingen, Germany). Prior to the measurements, the profilometer was calibrated according to the manufacturer’s instructions. The specimens were gently air-dried to remove excess surface moisture before evaluation. Surface roughness was expressed as the arithmetic mean roughness (Ra, µm). Measurements were performed using a cut-off length of 0.25 mm and an evaluation length of 4 mm. For each specimen, three consecutive measurements were obtained from randomly selected locations on the specimen surface, and the mean Ra value was calculated for statistical analysis. All measurements were performed by the same blinded examiner under standardized laboratory conditions (S.N.Y.).

### 2.9. Fracture-Load Testing

**Figure 2:**
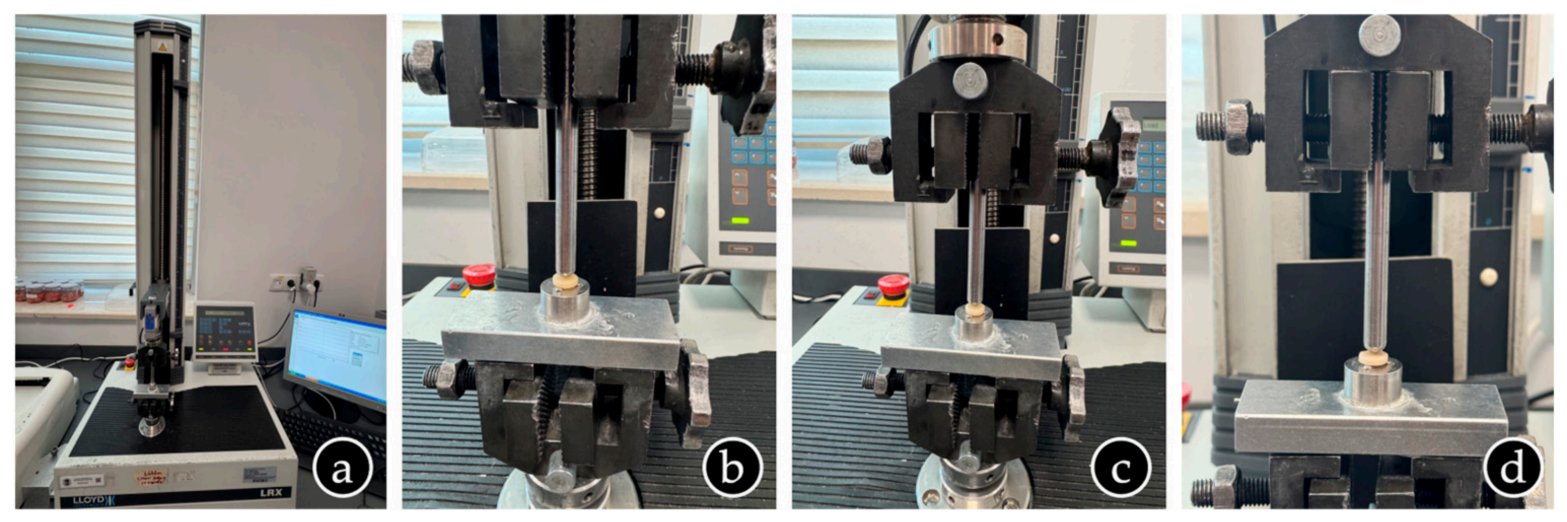
Universal testing machine setup used for fracture-load testing: (**a**) overall view of the testing configuration; close-up views showing the centrally positioned disc-shaped specimen and the 4 mm diameter stainless-steel cylindrical indenter aligned perpendicular to the specimen surface for (**b**) compomer (Dyract XP), (**c**) microfilled hybrid resin composite (G-ænial Posterior), and (**d**) glass hybrid restorative system (EQUIA Forte HT).

Fracture-load testing was performed using a universal testing machine (Lloyd Instruments LRX, Ametek, Fareham Hants, UK), following a previously published experimental protocol for disc-shaped restorative-material specimens [23]. Prior to testing, the universal testing machine was calibrated according to the manufacturer’s instructions. A load of 100 N was applied to a reference specimen, and the maximum permissible error was set at 0.05%. Each disc-shaped specimen was positioned centrally and horizontally on the testing platform. A 4 mm diameter stainless-steel cylindrical indenter was aligned perpendicular to the center of the specimen surface to ensure centrally directed compressive loading. The indenter was adjusted to establish stable contact with the specimen before loading. Compressive load was then applied continuously at a crosshead speed of 1 mm/min until the first visible fracture occurred. The maximum load recorded at the fracture event was expressed in Newtons (N). Fracture was initially identified visually during testing and subsequently confirmed using a stereomicroscope (Leica MZ21, Leica Microsystems GmbH, Wetzlar, Germany). All measurements were performed by the same blinded examiner under standardized laboratory conditions (A.D.). The actual universal testing machine configuration used for fracture-load testing is shown in Figure 2. Following fracture confirmation, fractured specimens were visually inspected to document their general fracture appearance. No formal fracture-mode classification or quantitative fracture-pattern analysis was performed.

### 2.10. Statistical Analysis

Statistical analyses were conducted using IBM SPSS Statistics for Windows, version 30.0 (IBM Corp., Armonk, NY, USA). Fracture-load and surface roughness were analyzed separately using three-way factorial general linear models, with restorative material, hemostatic agent, and thermocycling protocol entered as fixed factors. All main effects and two-way and three-way interactions were included in the models, and Type III sums of squares were used. Normality and homogeneity of variance were assessed using the Shapiro–Wilk and Levene tests, respectively. Fracture-load was analyzed on the original scale. Because surface roughness did not adequately satisfy the model assumptions, a natural logarithmic transformation was applied before analysis. Estimated marginal means, standard errors, and 95% confidence intervals were calculated. Pairwise comparisons were adjusted using the Bonferroni method. For surface roughness, model estimates were exponentiated and presented as geometric means and geometric mean ratios on the original micrometer scale. Significant interactions were further investigated using simple-effects analyses. All tests were two-sided, with statistical significance set at *p* < 0.05.

**Figure 3:**
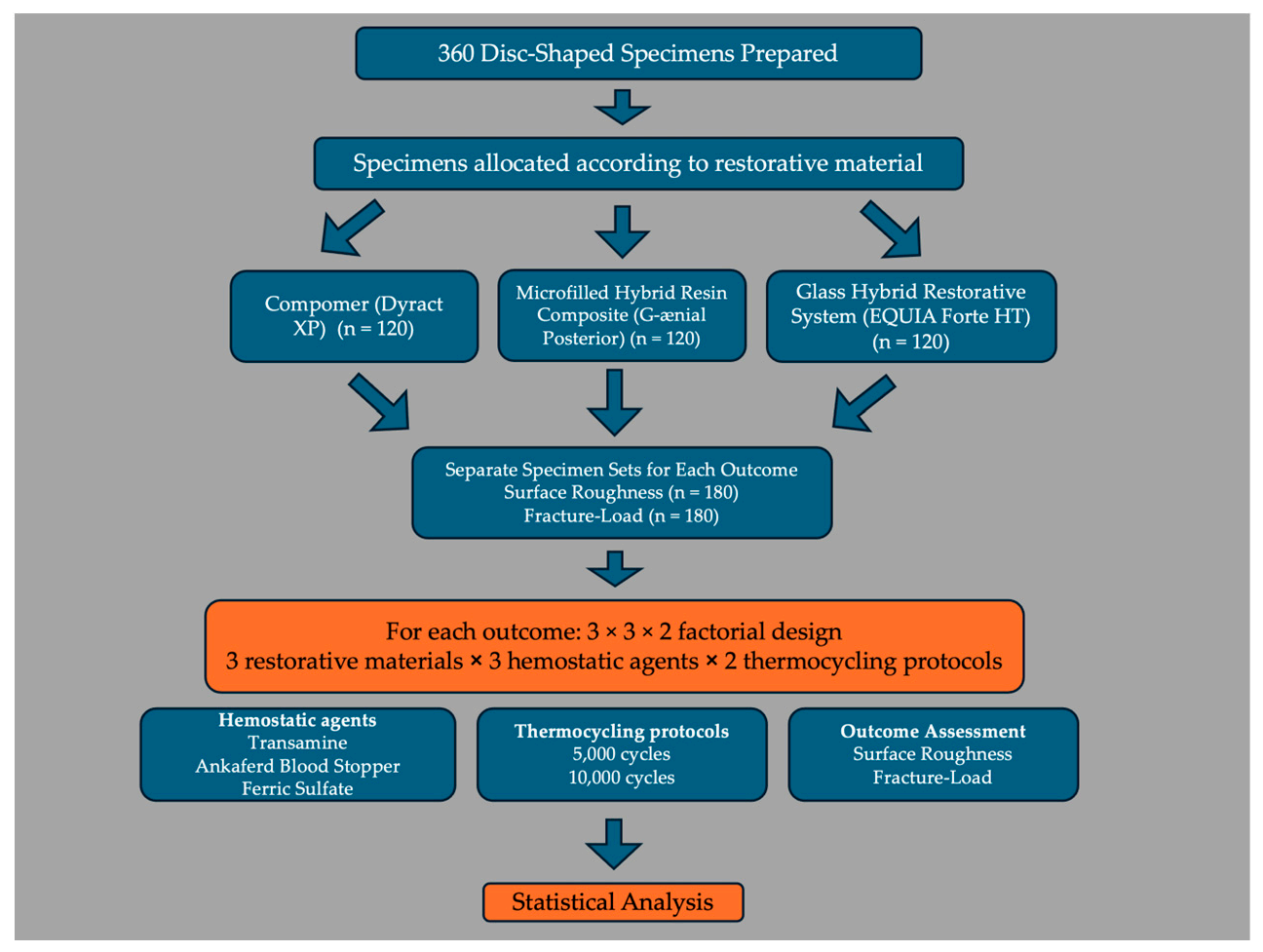
Methodological flow diagram of the experimental design, including preparation of disc-shaped specimens, grouping according to restorative material and hemostatic agent, artificial aging by thermocycling, surface roughness and fracture-load testing, and statistical analysis.

For a concise visual overview of the study design, the complete experimental workflow from specimen preparation to outcome assessment and statistical analysis is presented in Figure 3.

## 3. Results

**Figure 4:**
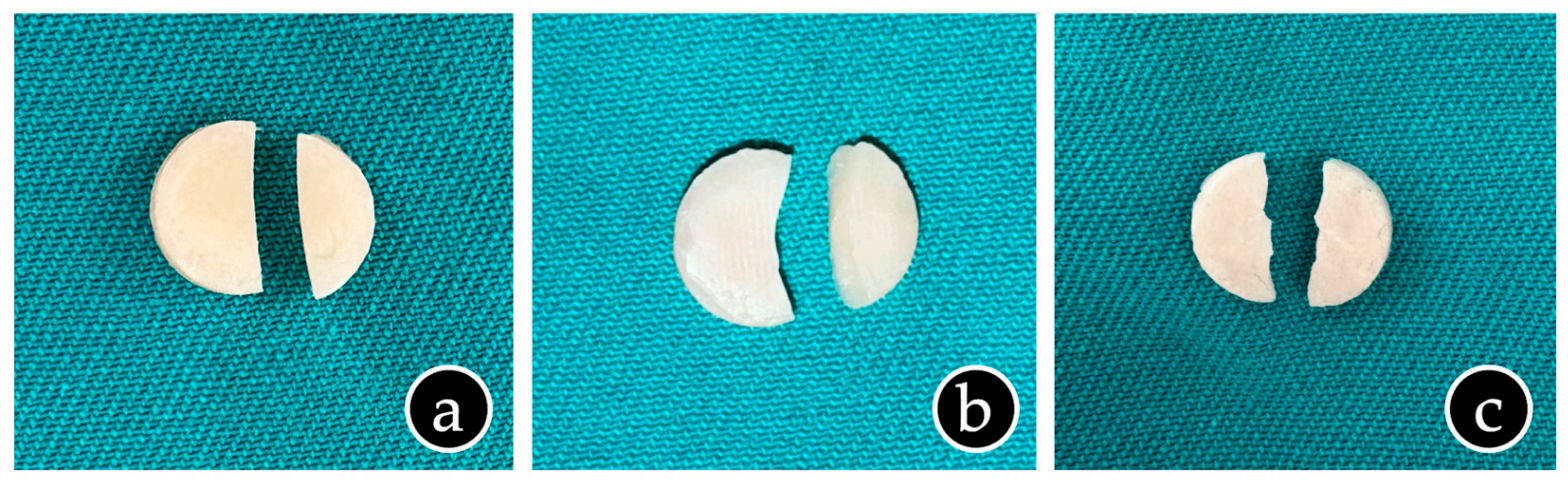
Representative fractured specimens following fracture-load testing: (**a**) compomer (Dyract XP); (**b**) microfilled hybrid composite resin (G-ænial Posterior); and (**c**) glass hybrid restorative system (EQUIA Forte HT). The images illustrate the general post-fracture appearance of the disc-shaped specimens after centrally applied compressive loading.

Following fracture-load testing, the fractured specimens were visually inspected to document their general post-fracture appearance. Representative examples from the three restorative materials are shown in Figure 4.

Descriptive statistics for fracture load and surface roughness according to restorative material, hemostatic agent, and thermocycling protocol are presented in Table 2. Across all experimental groups, mean fracture-load values ranged from 47.24 ± 5.02 N to 76.31 ± 4.59 N. The highest mean fracture load was recorded for composite specimens exposed to Transamine and subjected to 5000 thermocycles, whereas the lowest mean value was observed for glass hybrid specimens exposed to Ankaferd and subjected to 10,000 thermocycles. Within each restorative material–hemostatic agent combination, specimens subjected to 10,000 thermocycles generally showed lower mean fracture-load values than the corresponding specimens subjected to 5000 thermocycles. Surface roughness values ranged from 0.166 ± 0.050 µm to 0.443 ± 0.153 µm. The lowest mean surface roughness value was recorded for composite specimens exposed to Transamine and subjected to 5000 thermocycles, whereas the highest mean value was observed for glass hybrid specimens exposed to ferric sulfate and subjected to 10,000 thermocycles. Across all restorative material–hemostatic agent combinations, specimens subjected to 10,000 thermocycles showed numerically higher mean surface roughness values than the corresponding specimens subjected to 5000 thermocycles. These descriptive findings represent between-group differences at the end of the experimental protocols and should not be interpreted as within-specimen changes attributable to hemostatic-agent exposure.

**Figure 5:**
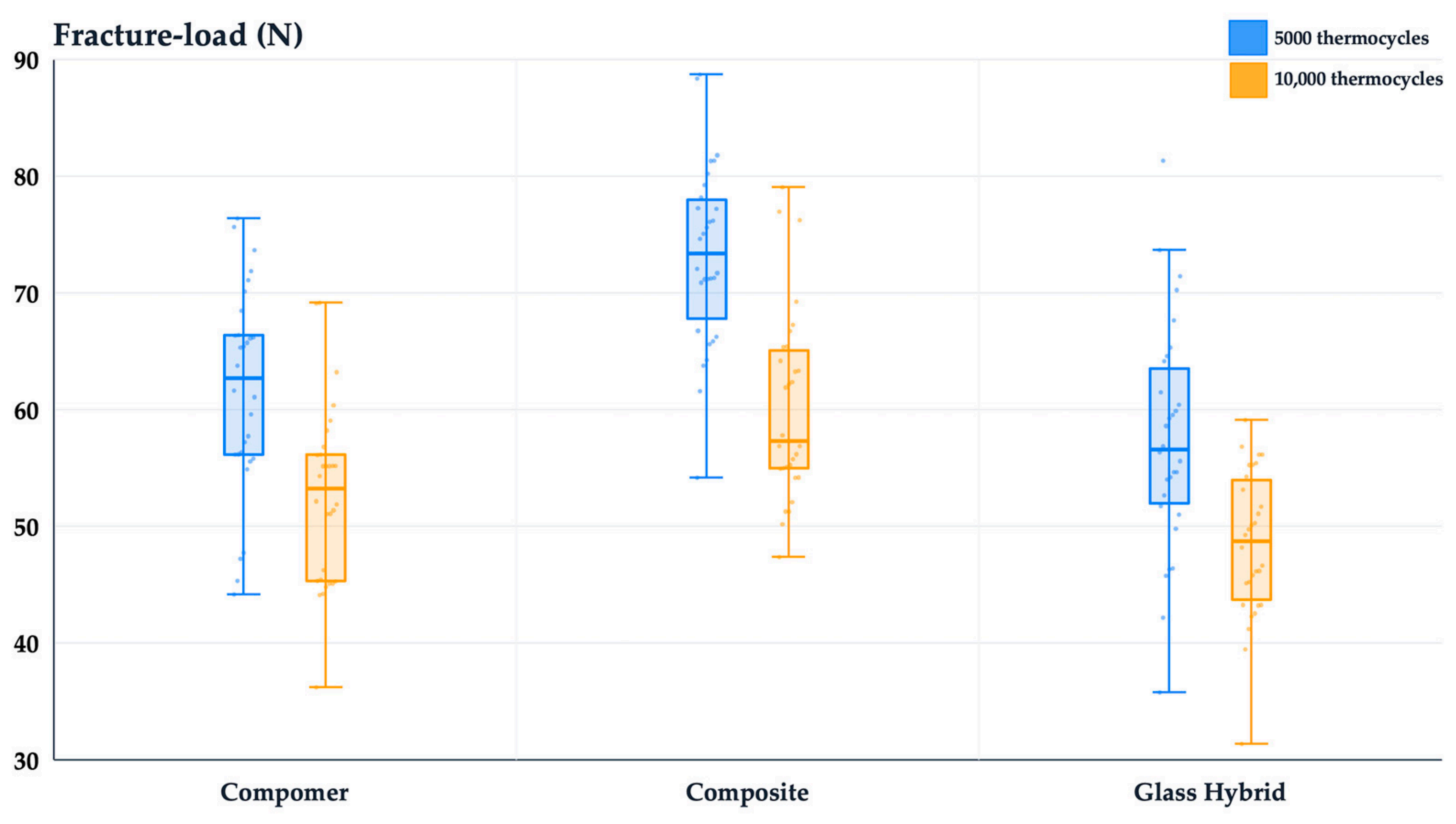
Distribution of fracture-load values according to restorative material and thermocycling protocol. Box plots present the median, interquartile range (IQR), whiskers (1.5 × IQR) and individual observations. Data are pooled across the three hemostatic-agent groups (*n* = 30 per box). Fracture-load values are presented descriptively.

Figure 5 presents the distribution of fracture-load values according to restorative material and thermocycling protocol. Across both thermocycling protocols, composite specimens exhibited the highest fracture-load distribution, whereas glass hybrid specimens showed the lowest distribution. Within each restorative material, specimens subjected to 10,000 thermocycles exhibited lower fracture-load values than those subjected to 5000 thermocycles. The variability in the measurements and the distribution of individual observations are illustrated by box plots (Figure 5).

**Figure 6:**
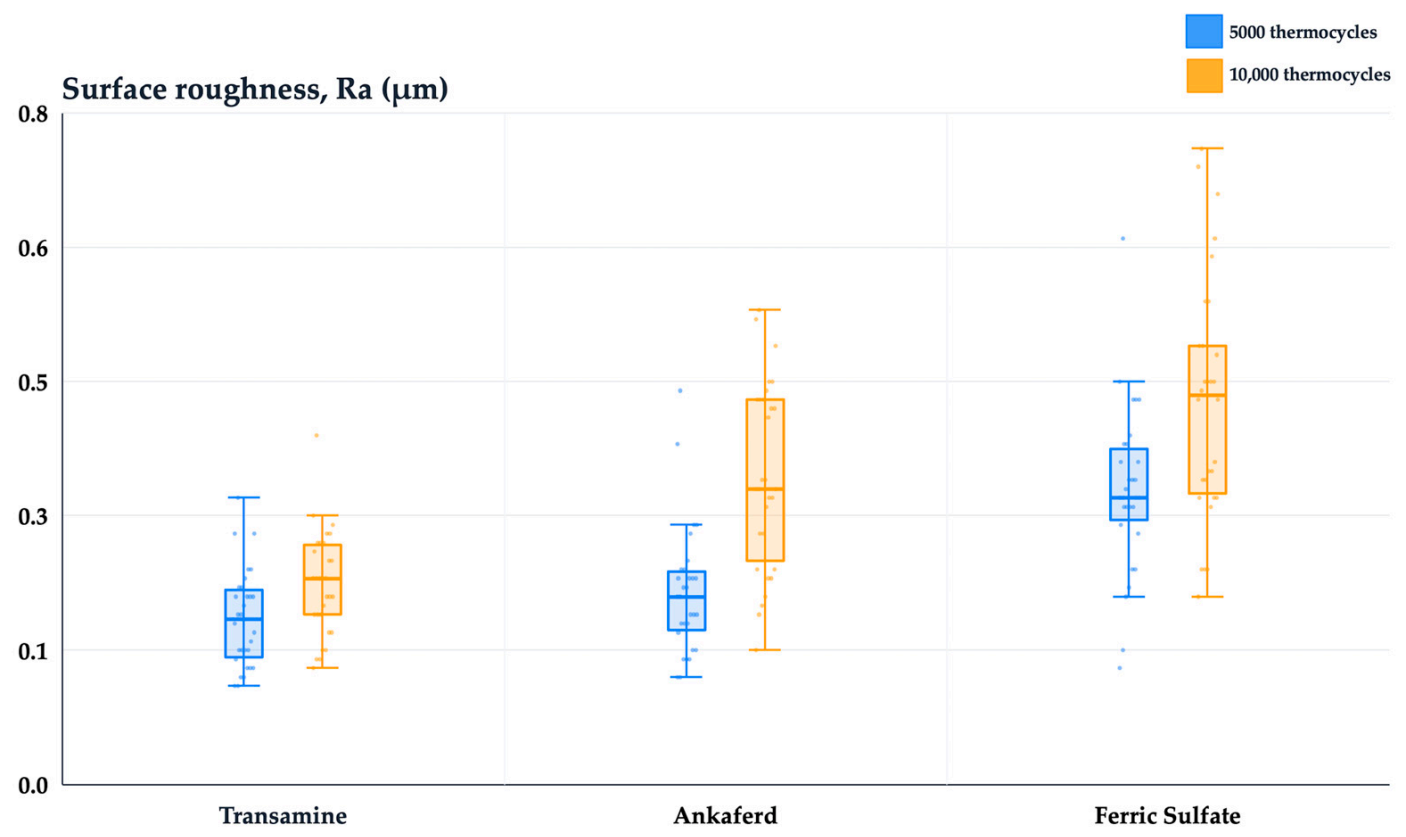
Distribution of surface roughness values according to hemostatic agent and thermocycling protocol. Box plots show the median, interquartile range (IQR), whiskers (1.5 × IQR) and individual observations. Data are pooled across the three restorative-material groups (*n* = 30 per box). Surface roughness values are presented descriptively.

Figure 6 shows the distribution of surface roughness values according to the hemostatic agent and artificial aging cycles. Across both cycles, specimens exposed to Transamine exhibited the lowest surface roughness distribution, whereas those exposed to ferric sulfate showed the highest distribution. Ankaferd demonstrated intermediate surface roughness values between the other two hemostatic agents. Within each hemostatic-agent group, specimens subjected to 10,000 thermocycles exhibited higher surface roughness values than those subjected to 5000 thermocycles. The variability in the measurements and the distribution of individual observations are illustrated by box plots (Figure 6).

**Figure 7:**
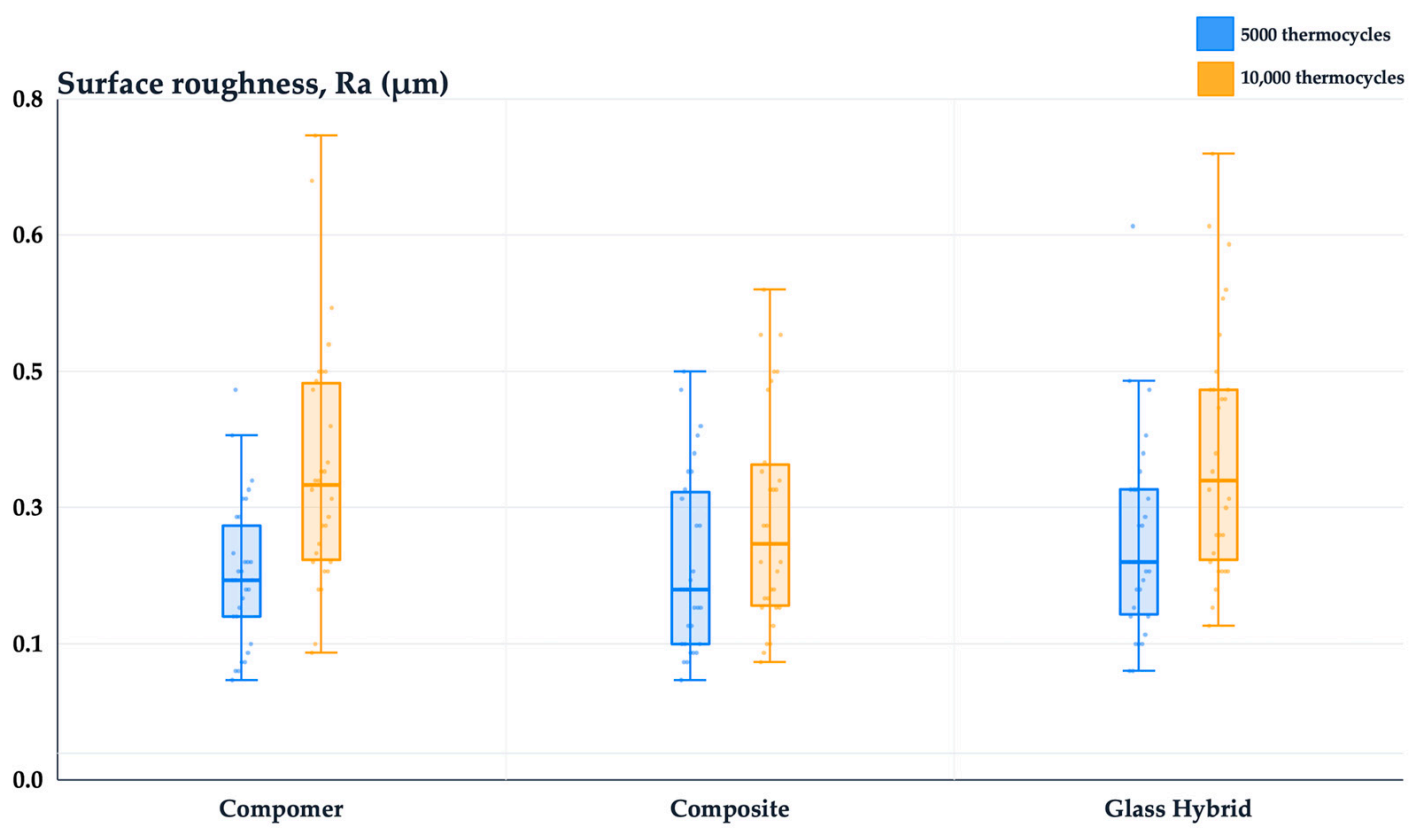
Distribution of surface roughness values according to restorative material and thermocycling protocol. Box plots show the median, interquartile range (IQR), whiskers (1.5 × IQR) and individual observations. Data are pooled across the three hemostatic-agent groups (*n* = 30 per box). Surface roughness values are presented descriptively.

Figure 7 shows the distribution of surface roughness values according to restorative material and thermocycling protocol. Across both thermocycling protocols, composite specimens exhibited the lowest surface roughness distribution, whereas glass hybrid specimens showed the highest distribution. Compomer demonstrated intermediate surface roughness values between the other two restorative materials. Within each restorative material, specimens subjected to 10,000 thermocycles exhibited higher surface roughness values than those subjected to 5000 thermocycles. The variability in the measurements and the distribution of individual observations are illustrated by box plots (Figure 7).

The estimated marginal means for fracture load are presented in Table 3. Among the restorative materials, composite exhibited the highest adjusted mean fracture load (66.81 N; 95% CI: 64.81–68.81), followed by compomer (57.14 N; 95% CI: 55.14–59.14) and glass hybrid (53.01 N; 95% CI: 51.01–55.01). Among the hemostatic-agent groups, Transamine and Ankaferd showed similar adjusted mean fracture-load values (60.83 N and 60.02 N, respectively), whereas the ferric sulfate group showed the lowest adjusted mean value (56.10 N; 95% CI: 54.10–58.10). With respect to the thermocycling protocol, the adjusted mean fracture load was higher in specimens subjected to 5000 thermocycles (64.18 N; 95% CI: 62.55–65.81) than in those subjected to 10,000 thermocycles (53.79 N; 95% CI: 52.16–55.42). Formal statistical comparisons of these estimated marginal means are presented in the subsequent tables.

Bonferroni-adjusted pairwise comparisons of the estimated marginal fracture-load means are presented in Table 4. Composite exhibited significantly higher estimated marginal fracture-load values than both compomer (mean difference = 9.67 N, 95% CI: 6.21–13.14, adjusted *p* < 0.001) and glass hybrid (mean difference = 13.80 N, 95% CI: 10.34–17.26, adjusted *p* < 0.001). In addition, compomer showed significantly higher estimated marginal fracture-load values than glass hybrid (mean difference = 4.13 N, 95% CI: 0.67–7.59, adjusted *p* = 0.013).

Bonferroni-adjusted pairwise comparisons of the estimated marginal fracture-load means according to the hemostatic agent are presented in Table 5. No significant difference was observed between the Transamine and Ankaferd groups (mean difference = 0.81 N, 95% CI: −2.65 to 4.27, adjusted *p* = 1.000). In contrast, the Transamine group exhibited significantly higher estimated marginal fracture-load values than the ferric sulfate group (mean difference = 4.73 N, 95% CI: 1.27–8.20, adjusted *p* = 0.003). Likewise, the Ankaferd group showed significantly higher estimated marginal fracture-load values than the ferric sulfate group (mean difference = 3.93 N, 95% CI: 0.46–7.39, adjusted *p* = 0.020).

Model-based comparisons of the estimated marginal fracture-load means between the two thermocycling protocols are presented in Table 6. Specimens subjected to 5000 thermocycles exhibited significantly higher estimated marginal fracture-load values than those subjected to 10,000 thermocycles (mean difference = 10.39 N, 95% CI: 8.08–12.70, adjusted *p* < 0.001).

Back-transformed estimated marginal geometric means for surface roughness are presented in Table 7. Among the restorative materials, glass hybrid showed the highest adjusted geometric mean surface roughness (0.287 µm; 95% CI: 0.266–0.310), followed by compomer (0.262 µm; 95% CI: 0.243–0.282) and composite (0.241 µm; 95% CI: 0.224–0.260). Among the hemostatic-agent groups, ferric sulfate showed the highest adjusted geometric mean surface roughness (0.358 µm; 95% CI: 0.332–0.386), followed by Ankaferd (0.258 µm; 95% CI: 0.240–0.278), whereas Transamine showed the lowest value (0.196 µm; 95% CI: 0.182–0.211). With respect to the thermocycling protocol, specimens subjected to 10,000 thermocycles showed a higher adjusted geometric mean surface roughness (0.306 µm; 95% CI: 0.288–0.326) than those subjected to 5000 thermocycles (0.225 µm; 95% CI: 0.212–0.239).

Bonferroni-adjusted pairwise comparisons of the adjusted geometric mean surface roughness values among the restorative materials are presented in Table 8. No significant differences were observed between compomer and composite (adjusted *p* = 0.374) or between compomer and glass hybrid (adjusted *p* = 0.256). However, composite exhibited significantly lower adjusted geometric mean surface roughness than glass hybrid (geometric mean ratio = 0.839, 95% CI: 0.737–0.955, adjusted *p* = 0.004).

All pairwise comparisons were statistically significant. The Transamine group exhibited significantly lower adjusted geometric mean surface roughness than both the Ankaferd group (geometric mean ratio = 0.759, 95% CI: 0.667–0.864, adjusted *p* < 0.001) and the ferric sulfate group (geometric mean ratio = 0.548, 95% CI: 0.481–0.624, adjusted *p* < 0.001). In addition, the Ankaferd group showed significantly lower adjusted geometric mean surface roughness than the ferric sulfate group (geometric mean ratio = 0.722, 95% CI: 0.634–0.822, adjusted *p* < 0.001) (Table 9).

Model-based comparisons of the adjusted geometric mean surface roughness values between the two thermocycling groups are presented in Table 10. Specimens subjected to 5000 thermocycles exhibited significantly lower adjusted geometric mean surface roughness than those subjected to 10,000 thermocycles (geometric mean ratio = 0.735, 95% CI: 0.674–0.801, adjusted *p* < 0.001) (Table 10). A significant hemostatic agent × thermocycling interaction was observed for surface roughness (F(2, 162) = 3.46, *p* = 0.034). Therefore, simple-effects analyses were performed to examine differences between hemostatic agents within each thermocycling protocol and between thermocycling protocols within each hemostatic-agent group.

Back-transformed estimated marginal geometric means for surface roughness according to the hemostatic agent × thermocycling interaction are presented in Table 11. Among specimens subjected to 5000 thermocycles, the adjusted geometric mean surface roughness values were 0.178 µm for Transamine, 0.204 µm for Ankaferd, and 0.314 µm for ferric sulfate. Among specimens subjected to 10,000 thermocycles, the corresponding adjusted geometric mean values were 0.216 µm, 0.326 µm, and 0.407 µm, respectively. Within each thermocycling group, Transamine showed the lowest adjusted geometric mean surface roughness, whereas ferric sulfate showed the highest. In addition, within each hemostatic-agent group, specimens subjected to 10,000 thermocycles exhibited higher adjusted geometric mean surface roughness values than those subjected to 5000 thermocycles (Table 11).

Among specimens subjected to 5000 thermocycles, no significant difference was observed between the Transamine and Ankaferd groups (adjusted *p* = 0.195). However, both the Transamine and Ankaferd groups exhibited significantly lower adjusted-geometric mean surface roughness than the ferric sulfate group (geometric mean ratios = 0.565 and 0.650, respectively; both adjusted *p* < 0.001). Among specimens subjected to 10,000 thermocycles, all pairwise comparisons were statistically significant. The Transamine group exhibited the lowest adjusted geometric mean surface roughness, followed by Ankaferd and ferric sulfate, with each comparison reaching statistical significance (all adjusted *p* ≤ 0.012) (Table 12).

Model-based simple-effects comparisons of the adjusted geometric mean surface roughness values between the 5000- and 10,000-thermocycle groups within each hemostatic-agent group are presented in Table 13. For all three hemostatic agents, specimens subjected to 5000 thermocycles exhibited significantly lower adjusted-geometric mean surface roughness than those subjected to 10,000 thermocycles. This difference was observed for the Transamine group (geometric mean ratio = 0.820, 95% CI: 0.706–0.952, *p* = 0.010), the Ankaferd group (geometric mean ratio = 0.626, 95% CI: 0.539–0.727, *p* < 0.001), and the ferric sulfate group (geometric mean ratio = 0.772, 95% CI: 0.665–0.897, *p* < 0.001) (Table 13).

## 4. Discussion

The principal findings of the present in vitro study showed significant differences in fracture-load and surface roughness values according to the restorative material, hemostatic agent, and thermocycling protocol. Composite resin exhibited the highest fracture-load values, whereas the glass hybrid restorative material showed the lowest fracture-load values and the numerically highest surface roughness value. Among the hemostatic-agent groups, specimens exposed to ferric sulfate showed the lowest fracture-load and highest surface roughness values, whereas those exposed to tranexamic acid showed the most favorable overall values. Specimens subjected to 10,000 thermocycles exhibited lower fracture-load and higher surface roughness values than those subjected to 5000 thermocycles. In addition, the significant hemostatic agent × thermocycling interaction for surface roughness indicated that differences among the hemostatic-agent groups varied according to the thermocycling regimen. Accordingly, the null hypotheses were rejected. However, because the study did not include an untreated control group, the findings demonstrate differences among hemostatic-agent-exposed groups rather than an increase or decrease caused by hemostatic-agent contact itself. Fracture-load testing was selected to provide a standardized comparative assessment of the load-bearing behavior of the restorative materials after hemostatic-agent exposure and artificial aging. Although this laboratory test does not fully reproduce the complex multidirectional loading conditions experienced by clinical restorations, it enables controlled comparison of the ability of different restorative materials to withstand compressive loading before fracture. In addition, evaluating fracture load together with surface roughness allowed complementary assessment of bulk mechanical behavior and surface characteristics within the same experimental framework.

Direct evidence regarding the effects of hemostatic agents on the fracture-load of restorative materials is scarce. Most previous studies have focused on their effects on dentin bonding, adhesive interfaces, or the dislocation resistance of restorative materials rather than on the intrinsic fracture behavior of the restorative material itself [28,29]. Within this limited evidence base, the present finding that ferric sulfate was associated with lower fracture-load than tranexamic acid and Ankaferd Blood Stopper is broadly consistent with studies reporting unfavorable effects of ferric sulfate contamination on adhesive performance. Although Hoorizad et al. [19] did not observe a statistically significant reduction in microshear bond strength following ferric sulfate contamination, bond strength consistently showed lower values across all tested adhesive systems after both 24 h and 3 months of water storage. The authors concluded that ferric sulfate contamination could negatively affect the bonding performance of both total-etch and self-etch adhesives, suggesting that this acidic hemostatic agent may interfere with adhesive interactions at the dentin surface [19]. Abu-Nawareg et al. [30] similarly demonstrated that dentin contamination with 20% ferric sulfate resulted in significantly lower shear bond strength than contamination with aluminum chloride, irrespective of the cleansing protocol applied. The authors attributed this finding to the different chemical composition and mechanism of action of ferric sulfate and further suggested that its higher viscosity, owing to its gel formulation, may hinder complete removal from the dentin surface. Although their study evaluated resin–dentin bond strength rather than the fracture strength of restorative materials, the consistently less favorable performance associated with ferric sulfate is in agreement with the present findings. Nevertheless, because the present study did not investigate the underlying mechanisms responsible for fracture failure, any relationship between ferric sulfate contamination and the mechanical behavior of restorative materials should be interpreted with caution and warrants further investigation.

The comparatively less favorable performance associated with ferric sulfate may be related to its acidic and protein-precipitating mechanism of action. Bandi et al. [7] reported that ferric sulfate achieves hemostasis through the formation of ferric–protein complexes under acidic conditions, whereas Yılmaz et al. [31] demonstrated that ferric sulfate contamination may result in persistent iron-associated surface deposits and qualitative surface alterations on dentin, depending on the subsequent conditioning protocol [7,31]. Although these findings were obtained from dentin rather than restorative materials, they suggest that ferric sulfate contamination may leave persistent surface residues capable of influencing subsequent material behavior. Such residues could potentially facilitate surface defects or crack initiation during mechanical loading and thermocycling; however, this explanation should be regarded as a mechanistic hypothesis because the present study did not include microscopic or chemical surface characterization. Consequently, the lower fracture-load associated with ferric sulfate exposure in the present study should be interpreted as an observation that is consistent with, but does not directly confirm, the mechanisms proposed in previous bond-strength and surface-characterization studies.

In contrast, tranexamic acid was associated with the most favorable fracture-load outcomes among the tested hemostatic agents. To the best of our knowledge, no previous study has investigated the direct effect of tranexamic acid on the fracture-load of restorative materials, precluding direct comparison with the present findings. Unlike ferric sulfate, tranexamic acid is an antifibrinolytic agent that stabilizes the developing clot by inhibiting plasminogen activation and fibrin degradation rather than by inducing acidic protein precipitation. Its effectiveness as a topical hemostatic agent has been well documented, particularly in oral surgery and in patients receiving anticoagulant therapy [32,33]. Therefore, the comparatively higher fracture-load values associated with tranexamic acid in the present study may be related to its distinct mechanism of hemostatic action. Nevertheless, further investigations incorporating surface chemical and microscopic analyses are required to determine whether tranexamic acid interacts directly with restorative material surfaces or whether the comparatively favorable fracture-load values observed in the present study are related to its distinct mechanism of hemostatic action.

Ankaferd Blood Stopper demonstrated an intermediate effect on fracture-load, with significantly higher fracture-load values than ferric sulfate but values comparable to those of tranexamic acid. To the best of our knowledge, no previous study has directly evaluated the influence of Ankaferd on the fracture-load of restorative materials, precluding direct comparison with the present findings. Most available studies have instead focused on its influence on adhesive performance. Arslan et al. [34] evaluated the effect of Ankaferd contamination on the shear bond strength of total-etch and self-etch adhesive systems and reported that Ankaferd contamination reduced the bond strength of all tested adhesive systems [34]. However, because bond strength and fracture strength represent different mechanical properties, direct comparison with the present findings should be interpreted with caution. Ankaferd achieves hemostasis through the rapid formation of an encapsulated protein network that provides focal points for erythrocyte aggregation without affecting individual coagulation factors [35]. Although the hemostatic mechanism of Ankaferd differs fundamentally from the acidic protein-precipitating mechanism of ferric sulfate, it remains unclear whether this difference is related to the fracture-load values observed in the present study. Further studies combining fracture-load testing with surface characterization and chemical analyses are warranted to determine whether the unique hemostatic mechanism of Ankaferd is associated with different interactions with restorative material surfaces.

Compared with fracture-load, considerably fewer studies have investigated the influence of hemostatic agents on the surface roughness of restorative materials. Most previous studies have primarily focused on the effects of hemostatic contamination on adhesive performance, bond strength, or dentin surface morphology, whereas evidence regarding alterations in the surface characteristics of restorative materials remains scarce. In the present study, ferric sulfate was associated with the highest surface roughness values, followed by Ankaferd Blood Stopper, whereas tranexamic acid exhibited the lowest surface roughness values. Furthermore, the significant interaction between the hemostatic agent and thermocycling protocol indicated that the differences among the hemostatic-agent groups varied according to the thermocycling protocol.

Among the tested hemostatic agents, ferric sulfate exhibited the highest surface roughness values. Although no previous study has directly evaluated the effect of ferric sulfate on the surface roughness of restorative materials, previous investigations on dentin have shown that ferric sulfate contamination may alter surface morphology and leave residual iron-associated deposits following subsequent conditioning procedures [31]. Bandi et al. [7] reported that ferric sulfate achieves hemostasis through its acidic, protein-precipitating action, leading to the formation of ferric–protein complexes at the application site. Furthermore, Yılmaz et al. [31] observed persistent iron-associated surface deposits together with protocol-dependent surface morphological features on ferric sulfate-contaminated dentin using scanning electron microscopy (SEM) and (energy-dispersive X-ray spectroscopy) EDS analyses. These observations may partly explain the higher surface roughness values associated with ferric sulfate in the present study. However, because neither surface morphology nor chemical composition was directly evaluated, the mechanisms underlying these findings remain speculative. Further studies combining surface roughness measurements with scanning electron microscopy, elemental analysis, and surface chemical analyses are warranted to clarify how ferric sulfate interacts with restorative material surfaces.

The response of restorative materials to hemostatic-agent exposure may also depend on their chemical composition and matrix–filler architecture. In the present study, the microfilled hybrid resin composite contained a methacrylate-based organic matrix with pre-polymerized and fluoro-alumino-silicate fillers, whereas the compomer combined a dimethacrylate resin matrix with acid-functional monomers and ion-releasing glass fillers, and the glass hybrid restorative system was primarily based on fluoro-alumino-silicate glass and polyacrylic-acid chemistry. Previous studies have shown that acidic environments can promote hydrolytic degradation of resin matrices and weaken the filler–matrix interface, thereby reducing mechanical properties and increasing susceptibility to surface degradation [36]. In addition, chemical gingival displacement agents, including ferric sulfate, have been shown to affect the physicochemical behavior of dental materials after direct contact [37]. Therefore, the comparatively lower fracture-load and higher surface roughness values observed in some compomer and glass hybrid groups after ferric sulfate exposure may reflect, at least in part, differences in material chemistry and susceptibility to acidic conditions. However, because no direct chemical, microscopic, or interfacial analyses were performed in the present study, this interpretation should be considered mechanistic and hypothesis-generating rather than confirmatory.

The present study has several strengths. To the best of our knowledge, it is among the first studies to directly compare fracture-load and surface roughness values of contemporary restorative materials following exposure to different topical hemostatic agents and thermocycling regimens. Unlike previous investigations that have primarily focused on dentin bonding or adhesive performance, the present study evaluated the restorative materials themselves under standardized experimental conditions. The inclusion of three hemostatic agents with distinct mechanisms of action, three restorative materials representing different material classes, and two artificial-aging protocols enabled a broad factorial comparison of material-, agent- and thermocycling-related differences. In addition, separate specimen sets were used for the two outcome assessments, and all procedures and measurements were performed under standardized and blinded conditions. The standardized 20 s application period was intended to simulate an inadvertent chairside contact scenario. Finally, evaluating both fracture load and surface roughness within the same experimental framework provided complementary information regarding the load-bearing behavior and surface characteristics of the tested materials.

Several limitations of the present study should be acknowledged. First of all, this was an in vitro investigation, and thermocycling alone cannot fully reproduce the complex oral environment, including saliva, biofilm, pH fluctuations, enzymatic activity, and repeated occlusal loading. Although this comparative design was selected to address differences among the three hemostatic-agent-exposed groups, the absence of an untreated control remains an important limitation. Therefore, the findings permit comparisons among the three hemostatic-agent-exposed groups but do not establish whether hemostatic-agent contact itself increased or decreased fracture load or surface roughness relative to unexposed specimens. Baseline surface roughness was also not measured before agent application; consequently, within-specimen surface changes could not be quantified. Because fracture testing is destructive, before-and-after fracture measurements cannot be obtained from the same specimen; therefore, future studies should include parallel untreated and distilled-water-control groups, together with baseline measurements where feasible, to distinguish the absolute effects of hemostatic-agent exposure from those related to storage and artificial aging. In addition, only one 20 s exposure and one rinsing procedure were evaluated; thus, the findings may not apply to longer or repeated exposure, incomplete removal, or alternative decontamination procedures. Only three restorative materials and three hemostatic formulations were tested, which limits generalizability. Additionally, the study did not include microscopic, elemental, or chemical surface characterization; therefore, the mechanisms underlying the observed between-group differences remain hypothetical.

Future research should further clarify the interactions between topical hemostatic agents and restorative materials by combining mechanical testing with advanced surface characterization techniques, such as SEM, atomic force microscopy (AFM), EDS and surface chemical analyses. Incorporating baseline and post-exposure surface roughness measurements would also provide a more direct assessment of surface alterations following hemostatic contamination. In addition, future studies should investigate different contamination protocols, including varying exposure durations, repeated applications, and different removal procedures, to better reflect the diversity of clinical scenarios. Expanding the range of restorative materials and topical hemostatic agents, together with evaluating additional clinically relevant properties such as microhardness, wear resistance, gloss retention, color stability, ion release, and bond durability, would provide a more comprehensive understanding of material performance. Finally, long-term aging protocols that combine thermocycling with mechanical fatigue and, ultimately, well-designed clinical studies are needed to determine the clinical significance of the present findings and to establish evidence-based recommendations for the safe use of topical hemostatic agents during restorative procedures.

## 5. Conclusions

Within the limitations of this in vitro study, fracture-load and surface roughness values differed according to the restorative material, hemostatic agent, and thermocycling protocol. Composite resin exhibited the highest fracture-load values, whereas the glass hybrid restorative material exhibited the lowest fracture-load values and the numerically highest surface roughness values. Among the hemostatic-agent groups, ferric sulfate was associated with the lowest fracture-load and highest surface roughness values, while tranexamic acid showed the most favorable overall mechanical and surface characteristics. In addition, specimens subjected to 10,000 thermocycles exhibited lower fracture-load and higher surface roughness values than those subjected to 5000 thermocycles. These findings indicate that restorative materials exposed to different topical hemostatic agents may demonstrate different mechanical and surface characteristics under standardized experimental conditions. However, because an untreated control group was not included, the present findings should be interpreted as differences among hemostatic-agent-exposed groups rather than as evidence that hemostatic-agent contact itself increases or decreases fracture load or surface roughness. Further laboratory and clinical studies incorporating untreated controls and comprehensive surface characterization are needed to clarify the mechanisms and clinical relevance of these observations.

## Figures and Tables

**Table 1:** Composition and characteristics of the restorative materials evaluated in the study.

Restorative Material	Material Class	Manufacturer	Main Chemical Composition
Dyract XP	Polyacid-modified composite resin (compomer)	Dentsply DeTrey GmbH, Konstanz, Germany	Urethane dimethacrylate (UDMA), carboxylic-acid-modified dimethacrylate (TCB resin), triethylene glycol dimethacrylate (TEGDMA), trimethacrylate resin (TMPTMA), other dimethacrylate resins, camphorquinone, ethyl-4-(dimethylamino)benzoate, butylated hydroxytoluene (BHT), UV stabilizer, strontium-alumino-sodium-fluoro-phosphor-silicate glass, highly dispersed silicon dioxide, strontium fluoride, and pigments
G-ænial Posterior	Resin composite	GC Corporation, Tokyo, Japan	Methacrylate monomers, including UDMA and dimethacrylate co-monomers; pre-polymerized fillers containing silica and strontium/lanthanoid fluoride; fluoro-alumino-silicate glass; fumed silica; pigments and photoinitiator/catalyst system
EQUIA Forte HT	Glass hybrid restorative system	GC Corporation, Tokyo, Japan	EQUIA Forte HT Fil: powder—fluoro-alumino-silicate glass, polyacrylic acid, pigments; liquid—polyacrylic acid and carboxylic acid. EQUIA Forte Coat: multifunctional monomer, methacrylate, silicon dioxide, initiator, phosphoric-acid-ester monomer, and stabilizer

**Table 2:** Descriptive statistics for fracture load and surface roughness according to restorative material, hemostatic agent and thermocycling protocol.

Restorative Material	Hemostatic Agent	Thermocycles	n	Fracture-Load (N) Mean ± SD	Surface Roughness (µm) Mean ± SD
Compomer	Transamine	5000	10	63.78 ± 9.71	0.171 ± 0.046
Compomer	Transamine	10,000	10	51.76 ± 10.12	0.243 ± 0.072
Compomer	Ankaferd	5000	10	63.44 ± 7.56	0.215 ± 0.046
Compomer	Ankaferd	10,000	10	53.82 ± 7.62	0.367 ± 0.088
Compomer	Ferric sulfate	5000	10	57.78 ± 8.39	0.296 ± 0.084
Compomer	Ferric sulfate	10,000	10	52.25 ± 4.65	0.421 ± 0.165
Composite	Transamine	5000	10	76.31 ± 4.59	0.166 ± 0.050
Composite	Transamine	10,000	10	66.39 ± 7.64	0.189 ± 0.045
Composite	Ankaferd	5000	10	75.36 ± 9.14	0.192 ± 0.045
Composite	Ankaferd	10,000	10	59.91 ± 7.50	0.258 ± 0.069
Composite	Ferric sulfate	5000	10	68.33 ± 6.82	0.353 ± 0.068
Composite	Ferric sulfate	10,000	10	54.56 ± 4.33	0.420 ± 0.091
Glass Hybrid	Transamine	5000	10	56.63 ± 8.24	0.218 ± 0.056
Glass Hybrid	Transamine	10,000	10	50.14 ± 6.21	0.239 ± 0.041
Glass Hybrid	Ankaferd	5000	10	60.37 ± 10.97	0.236 ± 0.106
Glass Hybrid	Ankaferd	10,000	10	47.24 ± 5.02	0.404 ± 0.099
Glass Hybrid	Ferric sulfate	5000	10	55.62 ± 10.35	0.336 ± 0.121
Glass Hybrid	Ferric sulfate	10,000	10	48.06 ± 7.66	0.443 ± 0.153

**Table 3:** Estimated marginal means of fracture-load according to restorative material, hemostatic agent and thermocycling protocol.

**Restorative Material**	**Estimated Marginal Mean (N)**	**SE**	**95% CI lower**	**95% CI upper**
Compomer	57.14	1.01	55.14	59.14
Composite	66.81	1.01	64.81	68.81
Glass hybrid	53.01	1.01	51.01	55.01
**Hemostatic Agent**	**Estimated Marginal Mean (N)**	**SE**	**95% CI lower**	**95% CI upper**
Transamine	60.83	1.01	58.84	62.83
Ankaferd	60.02	1.01	58.03	62.02
Ferric sulfate	56.10	1.01	54.10	58.10
**Thermocycles**	**Estimated Marginal Mean (N)**	**SE**	**95% CI lower**	**95% CI upper**
5000 cycles	64.18	0.83	62.55	65.81
10,000 cycles	53.79	0.83	52.16	55.42

**Table 4:** Bonferroni-adjusted pairwise comparisons of estimated marginal mean fracture-load among the restorative materials.

Material I	Material J	Mean Difference (I − J), N	SE	Bonferroni 95% CI Lower	Bonferroni 95% CI Upper	t	Adjusted *p*
**Compomer**	Composite	−9.67	1.43	−13.14	−6.21	−6.76	<0.001
**Compomer**	Glass hybrid	4.13	1.43	0.67	7.59	2.88	0.013
**Composite**	Glass hybrid	13.80	1.43	10.34	17.26	9.64	<0.001

**Table 5:** Bonferroni-adjusted pairwise comparisons of estimated marginal mean fracture-load among the hemostatic agents.

Agent I	Agent J	Mean Difference (I − J), N	SE	Bonferroni 95% CI Lower	Bonferroni 95% CI Upper	t	Adjusted *p*
Transamine	Ankaferd	0.81	1.43	−2.65	4.27	0.57	1.000
Transamine	Ferric sulfate	4.73	1.43	1.27	8.20	3.31	0.003
Ankaferd	Ferric sulfate	3.93	1.43	0.46	7.39	2.74	0.020

**Table 6:** Model-based comparison of estimated marginal mean fracture-load between the 5000- and 10,000-thermocycle aging protocols.

Thermocycle I	Thermocycle J	Mean Difference (I − J), N	SE	95% CI Lower	95% CI Upper	t	*p*
**5000**	10,000	10.39	1.17	8.08	12.70	8.89	<0.001

**Table 7:** Back-transformed estimated marginal means of surface roughness according to restorative material, hemostatic agent, and thermocycling protocol.

**Restorative Material**	**Geometric mean (µm)**	**95% CI lower**	**95% CI upper**	**ln mean**	**SE (ln)**
Compomer	0.262	0.243	0.282	−1.340	0.038
Composite	0.241	0.224	0.260	−1.423	0.038
Glass hybrid	0.287	0.266	0.310	−1.248	0.038
**Hemostatic agent**	**Geometric mean (µm)**	**95% CI lower**	**95% CI upper**	**ln mean**	**SE (ln)**
Transamine	0.196	0.182	0.211	−1.630	0.038
Ankaferd	0.258	0.240	0.278	−1.354	0.038
Ferric sulfate	0.358	0.332	0.386	−1.028	0.038
**Thermocycles**	**Geometric mean (µm)**	**95% CI lower**	**95% CI upper**	**ln mean**	**SE (ln)**
5000 cycles	0.225	0.212	0.239	−1.491	0.031
10,000 cycles	0.306	0.288	0.326	−1.183	0.031

**Table 8:** Bonferroni-adjusted pairwise comparisons of surface roughness among restorative materials.

Material I	Material J	ln Difference (I − J)	Geometric Mean Ratio	Bonferroni 95% CI (Ratio)	t	Adjusted *p*
Compomer	Composite	0.083	1.086	0.954–1.237	1.54	0.374
Compomer	Glass hybrid	−0.093	0.911	0.801–1.038	−1.73	0.256
Composite	Glass hybrid	−0.175	0.839	0.737–0.955	−3.28	0.004

**Table 9:** Bonferroni-adjusted pairwise comparisons of surface roughness among hemostatic agents.

Agent I	Agent J	ln Difference (I − J)	Geometric Mean Ratio	Bonferroni 95% CI (Ratio)	t	Adjusted *p*
Transamine	Ankaferd	−0.276	0.759	0.667–0.864	−5.15	<0.001
Transamine	Ferric sulfate	−0.602	0.548	0.481–0.624	−11.23	<0.001
Ankaferd	Ferric sulfate	−0.326	0.722	0.634–0.822	−6.08	<0.001

**Table 10:** Comparison of surface roughness between the 5000 and 10,000 cycles.

Thermocycle I	Thermocycle J	ln Difference (I − J)	Geometric Mean Ratio	95% CI (Ratio)	t	*p*
5000	10,000	−0.308	0.735	0.674–0.801	−7.05	<0.001

**Table 11:** Back-transformed geometric estimated marginal means and 95% confidence intervals for surface roughness according to the hemostatic agent × thermocycling interaction.

Hemostatic Agent	Thermocycles	Geometric Mean (µm)	95% CI Lower	95% CI Upper	ln Mean	SE (ln)
Transamine	5000	0.178	0.160	0.197	−1.729	0.054
Ankaferd	5000	0.204	0.184	0.227	−1.588	0.054
Ferric sulfate	5000	0.314	0.283	0.350	−1.157	0.054
Transamine	10,000	0.216	0.195	0.241	−1.530	0.054
Ankaferd	10,000	0.326	0.294	0.363	−1.119	0.054
Ferric sulfate	10,000	0.407	0.366	0.453	−0.899	0.054

**Table 12:** Bonferroni-adjusted simple-effects comparisons of geometric mean surface roughness among hemostatic agents within each thermocycling protocol.

Thermocycles	Agent I	Agent J	ln Difference	Geometric Mean Ratio	Bonferroni 95% CI (Ratio)	Adjusted *p*
5000	Transamine	Ankaferd	−0.141	0.869	0.723–1.043	0.195
5000	Transamine	Ferric sulfate	−0.572	0.565	0.470–0.678	<0.001
5000	Ankaferd	Ferric sulfate	−0.431	0.650	0.541–0.781	<0.001
10,000	Transamine	Ankaferd	−0.411	0.663	0.552–0.796	<0.001
10,000	Transamine	Ferric sulfate	−0.632	0.532	0.443–0.639	<0.001
10,000	Ankaferd	Ferric sulfate	−0.221	0.802	0.668–0.963	0.012

**Table 13:** Model-based simple-effects comparisons of geometric mean surface roughness between 5000 and 10,000 thermocycles within each hemostatic agent group.

Hemostatic Agent	Thermocycle I	Thermocycle J	ln Difference	Geometric Mean Ratio	95% CI (Ratio)	*p*
Transamine	5000	10,000	−0.198	0.820	0.706–0.952	0.010
Ankaferd	5000	10,000	−0.469	0.626	0.539–0.727	<0.001
Ferric sulfate	5000	10,000	−0.258	0.772	0.665–0.897	<0.001

## Data Availability

The raw data supporting the conclusions of this article will be made available by the authors on request.

## Abbreviations

The following abbreviations are used in this manuscript:

CRIS	Checklist for reporting in vitro studies
SEM	Scanning electron microscopy
EDS	Energy-dispersive X-ray spectroscopy
AFM	Atomic force microscopy

## References

[1] Dhar V., Hsu K.L., Coll J.A., Ginsberg E., Ball B.M., Chhibber S., Johnson M., Kim M., Modaresi N., Tinanoff N. (2015). Evidence-Based Update of Pediatric Dental Restorative Procedures: Dental Materials. J. Clin. Pediatr. Dent..

[2] American Academy of Pediatric Dentistry (2017). Pediatric Restorative Dentistry. Pediatr. Dent..

[3] Dipalma G., Inchingolo A.M., Casamassima L., Nardelli P., Ciccarese D., De Sena P., Inchingolo F., Palermo A., Severino M., Maspero C.M.N. (2025). Effectiveness of Dental Restorative Materials in the Atraumatic Treatment of Carious Primary Teeth in Pediatric Dentistry: A Systematic Review. Children.

[4] Rodrigues J.A., Casagrande L., Araújo F.B., Lenzi T.L., Mariath A.A.S., Leal S.C., Takeshita E.M. (2019). Restorative Materials in Pediatric Dentistry. Pediatric Restorative Dentistry.

[5] BaniHani A., Santamaría R.M., Hu S., Maden M., Albadri S. (2022). Minimal Intervention Dentistry for Managing Carious Lesions into Dentine in Primary Teeth: An Umbrella Review. Eur. Arch. Paediatr. Dent..

[6] Alqahtani A.M., Alshihri Y.D., Alhumaid A.E., Al Nafaie M.M., Alnaim A.A. (2025). Advancements in Minimally Invasive Techniques in Pediatric Dentistry: A Review. Cureus.

[7] Bandi M., Mallineni S.K., Nuvvula S. (2017). Clinical Applications of Ferric Sulfate in Dentistry: A Narrative Review. J. Conserv. Dent..

[8] Sirohi K., Marwaha M., Gupta A., Bansal K., Srivastava A. (2017). Comparison of Clinical and Radiographic Success Rates of Pulpotomy in Primary Molars Using Ferric Sulfate and Bioactive Tricalcium Silicate Cement: An In Vivo Study. Int. J. Clin. Pediatr. Dent..

[9] Brar K.A., Kratunova E., Avenetti D., da Fonseca M.A., Marion I., Alapati S. (2021). Success of Biodentine and Ferric Sulfate as Pulpotomy Materials in Primary Molars: A Retrospective Study. J. Clin. Pediatr. Dent..

[10] Kazancıoğlu H.O., Cakır O., Ak G., Zülfikar B. (2013). The Effectiveness of a New Hemostatic Agent (Ankaferd Blood Stopper) for the Control of Bleeding Following Tooth Extraction in Hemophilia: A Controlled Clinical Trial. Turk. J. Haematol..

[11] Simsek C., Selek S., Koca M., Haznedaroglu I.C. (2017). Proteomic and Transcriptomic Analyses to Explain the Pleiotropic Effects of Ankaferd Blood Stopper. SAGE Open Med..

[12] Çiftçiler R., Haznedaroglu İ.C. (2020). Ankaferd Hemostat: From Molecules to Medicine. Turk. J. Med. Sci..

[13] Ciftciler R., Ciftciler A.E., Malkan U.Y., Haznedaroglu I.C. (2020). Pharmacobiological Management of Hemostasis within Clinical Backgrounds via Ankaferd Hemostat (Ankaferd Blood Stopper). SAGE Open Med..

[14] de Vasconcellos S.J., de Santana Santos T., Reinheimer D.M., Faria-E-Silva A.L., de Melo M.F., Martins-Filho P.R. (2017). Topical Application of Tranexamic Acid in Anticoagulated Patients Undergoing Minor Oral Surgery: A Systematic Review and Meta-Analysis of Randomized Clinical Trials. J. Craniomaxillofac. Surg..

[15] Mahardawi B., Jiaranuchart S., Arunjaroensuk S., Tompkins K.A., Somboonsavatdee A., Pimkhaokham A. (2023). The Effect of Different Hemostatic Agents Following Dental Extraction in Patients under Oral Antithrombotic Therapy: A Network Meta-Analysis. Sci. Rep..

[16] Bernades K.O., Hilgert L.A., Ribeiro A.P., Garcia F.C., Pereira P.N. (2014). The Influence of Hemostatic Agents on Dentin and Enamel Surfaces and Dental Bonding: A Systematic Review. J. Am. Dent. Assoc..

[17] Bourgi R., Cuevas-Suarez C.E., Devoto W., Monjarás-Ávila A.J., Monteiro P., Kharma K., Lukomska-Szymanska M., Hardan L. (2023). Effect of Contamination and Decontamination Methods on the Bond Strength of Adhesive Systems to Dentin: A Systematic Review. J. Esthet. Restor. Dent..

[18] Ebrahimi S.F., Shadman N., Abrishami A. (2013). Effect of Ferric Sulfate Contamination on the Bonding Effectiveness of Etch-and-Rinse and Self-Etch Adhesives to Superficial Dentin. J. Conserv. Dent..

[19] Hoorizad M., Heshmat H., Hosseini T.A., Kazemi S.S., Tabatabaei S.F. (2019). Effect of Hemostatic Agent on Microshear Bond Strength of Total-Etch and Self-Etch Adhesive Systems. Dent. Res. J..

[20] Bollen C.M., Lambrechts P., Quirynen M. (1997). Comparison of Surface Roughness of Oral Hard Materials to the Threshold Surface Roughness for Bacterial Plaque Retention: A Review of the Literature. Dent. Mater..

[21] Engel A.S., Kranz H.T., Schneider M., Tietze J.P., Piwowarcyk A., Kuzius T., Arnold W., Naumova E.A. (2020). Biofilm Formation on Different Dental Restorative Materials in the Oral Cavity. BMC Oral Health.

[22] Al-Sabur R., Kubit A., Khalaf H.I., Jurczak W., Dzierwa A., Korzeniowski M. (2023). Analysis of Surface Texture and Roughness in Composites Stiffening Ribs Formed by SPIF Process. Materials.

[23] Durmaz A.A., Demirel A., Ökte Z. (2025). The Evaluation of the Effects of Saliva Contamination on Microhardness and Fracture Strength of Different Aged Restorative Materials. J. Clin. Pediatr. Dent..

[24] Ntovas P., Korkut B., Loumprinis N., Rachiotis I., Rahiotis C. (2025). Influence of Composite Polishing Pastes on Surface Roughness and Their Stability After Simulated Tooth Brushing. Dent. J..

[25] Dutra D., Pereira G., Kantorski K.Z., Valandro L.F., Zanatta F.B. (2018). Does Finishing and Polishing of Restorative Materials Affect Bacterial Adhesion and Biofilm Formation? A Systematic Review. Oper. Dent..

[26] Gale M.S., Darvell B.W. (1999). Thermal Cycling Procedures for Laboratory Testing of Dental Restorations. J. Dent..

[27] Krithikadatta J., Gopikrishna V., Datta M. (2014). CRIS Guidelines (Checklist for Reporting In Vitro Studies): A Concept Note on the Need for Standardized Guidelines for Improving Quality and Transparency in Reporting In Vitro Studies in Experimental Dental Research. J. Conserv. Dent..

[28] Mempel C.A., Jacker-Guhr S., Lührs A.K. (2022). Contamination of Dentin with Hemostatic Agents—Is EDTA a Valuable Decontaminant before Using a Self-Etch Universal Adhesive?. J. Adhes. Dent..

[29] Pratabsingha J., Noppawong S., Thamsoonthorn C., Vichathai W., Saikaew P. (2023). Bonding Protocols to Reverse the Bond Strength of a Universal Adhesive to Hemostatic Agent-Contaminated Dentin. Oper. Dent..

[30] Abu-Nawareg M.M., Hajjaj M.S., AbuHaimed T.S., Ajaj R.A., Abuljadayel R., AlNowailaty Y., Alnoury A., Khouja N., Naguib G.H., Alzahrani S.J. (2024). The Effect of Hemostatic Agents and Dentin Cleansing Protocols on Shear Bond Strength of Resin Composite Using Universal Adhesive: An In Vitro Study. BMC Oral Health.

[31] Yılmaz N.A. (2026). Gallic Acid as a Biointeractive Dentin Conditioning Agent for Universal Adhesives: Bond Strength, Iron Retention, and Surface Characterization Following Ferric Sulfate Hemostatic Contamination. Clin. Oral Investig..

[32] Ng W., Jerath A., Wąsowicz M. (2015). Tranexamic Acid: A Clinical Review. Anaesthesiol. Intensive Ther..

[33] Picetti R., Shakur-Still H., Medcalf R.L., Standing J.F., Roberts I. (2019). What Concentration of Tranexamic Acid Is Needed to Inhibit Fibrinolysis? A Systematic Review of Pharmacodynamics Studies. Blood Coagul. Fibrinolysis.

[34] Arslan S., Ertaş H., Zorba Y.O. (2012). Influence of Ankaferd Blood Stopper on Shear Bond Strength of Bonding Systems. Dent. Mater. J..

[35] Goker H., Haznedaroglu I.C., Ercetin S., Kirazli S., Akman U., Ozturk Y., Firat H.C. (2008). Haemostatic Actions of the Folkloric Medicinal Plant Extract Ankaferd Blood Stopper. J. Int. Med. Res..

[36] Brânzan R.C., Tărăboanță I., Ghiorghe C.A., Stoleriu S., Cârlescu V., Tărăboanță-Gamen A.C., Andrian S. (2024). Evaluation of the Mechanical Properties of Different Dental Resin-Based Materials After Submersion in Acidic Beverages. Dent. J..

[37] Nowakowska D., Raszewski Z., Ziętek M., Saczko J., Kulbacka J., Więckiewicz W. (2018). The Setting Time of Polyether Impression Materials after Contact with Conventional and Experimental Gingival Margin Displacement Agents. J. Prosthodont..

